# Unusual presentation of Rosai-Dorfman disease in a 14-month-old Italian child: a case report and review of the literature

**DOI:** 10.1186/s12887-016-0595-9

**Published:** 2016-05-03

**Authors:** Francesco di Dio, Ilaria Mariotti, Elena Coccolini, Patrizia Bruzzi, Barbara Predieri, Lorenzo Iughetti

**Affiliations:** Pediatric Unit, Department of Medical and Surgical Sciences for Mothers, Children and Adults, University of Modena & Reggio Emilia, Via del Pozzo, 71, 41124 Modena, Italy

**Keywords:** Rosai-Dorfman disease, Histiocytosis, Lymphadenopathy, Parotid swelling, Steroid therapy

## Abstract

**Background:**

Rosai-Dorfman disease (RDD) is a rare form of histiocytosis characterized by histiocyte proliferation within lymph nodes and extranodal tissue. Here we report an unusual presentation of RDD in an Italian toddler. Moreover, we reviewed the pediatric case reports published between 2004 and 2014, focusing in particular on medical therapy.

**Case presentation:**

We report the case of a 14-month-old child who developed a progressive swelling of the right parotid, associated with systemic symptoms and abnormal blood tests. During diagnostic work-up, cervical, intraparotid, and unilateral hilar lymphadenopathies were found. Histopathological and immunohistochemistry studies of a cervical lymph node biopsy established the diagnosis of RDD, with positive PCR for Epstein - Barr virus on the biopsy specimen. Oral steroid therapy was started with progressive reduction in size of all lesions, resolution of systemic symptoms, and normalization of blood tests.

**Conclusion:**

RDD is generally considered a benign and self-limiting form of histiocytosis, usually associated with favorable prognosis. However, complications are not infrequent and fatal cases were reported even in children. Efforts should be made to establish the best therapeutic strategy for this disease, as no well-defined guidelines exist. Finally, RDD should be included in differential diagnosis of lymphadenopathy and parotid swelling even in very young children.

**Electronic supplementary material:**

The online version of this article (doi:10.1186/s12887-016-0595-9) contains supplementary material, which is available to authorized users.

## Background

Rosai-Dorfman disease (RDD), also known as sinus histiocytosis with massive lymphadenopathy, is a form of class II histiocytosis typically characterized by massive bilateral and painless cervical lymphadenopathy associated with systemic symptoms such as fever and weight loss. This is the most typical presentation, however, few diseases can present with such a diversity of signs and symptoms as RDD.

It is possible to distinguish two clinical forms of the disease: a systemic form, which the previous definition refers to, and an exclusively cutaneous one, sharing the same histopathological pattern as the “classical” form, but with different epidemiological characteristics, and with no involvement of tissues other than the skin [1, 2].

The systemic form, first described by Destombes in 1965, was recognized as a specific pathological entity in 1969 by Juan Rosai and Ronald Dorfman [3].

It is a rare disease, having a reported prevalence of 1:200.000 [4]. The disease affects mainly males (58 % vs 42 %) [5]. It is more frequent in subjects of African descent and in young adults, even if cases in individuals ranging from 1 to 74 years of age were reported [4]. The cutaneous form is even rarer, accounting for 3 % of the total cases. Furthermore, it appears to be more frequent in females (2 to 1 ratio) and in adults (there are no reported cases in children under 15 years of age) of Asian ethnicity [1, 2].

We describe an unusual presentation of systemic RDD in an Italian toddler. Moreover, we analyze recent case reports published in literature, focusing in particular on medical therapy of RDD.

## Case presentation

A 14-month-old child first presented to medical care at an outside Hospital for a swelling of the right parotid, without any other signs or symptoms. The swelling was noted by parents 1 month before, becoming more evident during the last 10 days. Past clinical and family histories were unremarkable. Clinical examination revealed the presence of a hard, painless swelling in the right parotid area, a palpable bilateral cervical lymphadenopathy (more evident on the right side of the neck), and a mild upper respiratory tract infection. The palpable lymph nodes were described as mobile and painless, having a maximum diameter of approximately 2 centimeters. First-line blood tests were negative, with no increase in C-reactive protein (CRP) or leukocytosis. Mononuclear spot test for Epstein - Barr virus (EBV) and other serological blood tests allowed us to exclude ongoing infections. Right parotid gland’s ultrasonography (US) showed cystic and solid nodular lesions with diameters between 6.5 mm and 24.6 mm. Similar formations were also noted within the left parotid, although of smaller dimensions. Bilateral cervical lymphadenopathy was confirmed by ultrasound, especially on the right side of the neck, with maximum lymph node diameter of 15 mm. The patient was discharged with oral antibiotic therapy (Amoxicillin plus Clavulanic acid). After 11 days, the child was referred to our Pediatric Department. Parents reported the onset of low-grade fever, an increased swelling and local pain. Blood tests were repeated, showing mild neutrophilic leukocytosis (white blood cells 14.61 × 10^9^/L, 68 % neutrophils), high erythrocyte sedimentation rate (ESR; 77 mm/hr), and CRP of 9.14 mg/dl (upper normal limit 0.8 mg/dl). Asides from the leukocytosis, full blood count was normal. US showed an enlargement of the previously reported lymph nodes in the right parotid, indicative for lymph node conglomerates (maximum diameter 40 mm).

Suspecting a bacterial infection, a broad-spectrum intravenous antibiotic was started, as well as Ibuprofen as anti-inflammatory therapy. Extended serological analysis were performed, including those for Mycoplasma pneumoniae, Toxoplasmosis, EBV, Cytomegalovirus (CMV), rubella virus, mumps virus, measles virus, type 1 Herpes simplex virus, human Herpesvirus 6 (HHV-6), varicella-zoster virus, Bartonella henselae, Adenovirus, Enterovirus and Parvovirus B19. All of these resulted negative for ongoing infections. However, an interesting finding was that EBNA-IgG for EBV were negative, with highly positive VCA IgG (121 U/ml; laboratory cut-off at 11,5 U/ml) and negative IgM, suggesting a recent infection. Blood PCR for EBV was not performed. HIV and tuberculosis tests were negative. Protein electrophoresis was normal.

Magnetic resonance imaging (MRI) revealed a 43 × 28 mm diameter solid mass attributable to lymph nodes, entirely occupying the right parotid gland. Gadolinium enhancement was irregular because of the presence of necrotic areas within the lesion. A lymph node conglomerate was present under the mass, having maximum extent of 6 cm.

Abdominal US and bone marrow aspiration were performed and were unremarkable.

Chest radiograph showed a right mediastinal enlargement. A cervical lymph node biopsy was then performed.

Quantitative PCR for EBV on the biopsy specimen was positive, with 802 copies/100.000 cells.

Histopathologic examination showed the presence of emperipolesis, with notable sinus infiltration of large histiocytic cells with pale cytoplasm. On immunohistochemistry, these cells displayed a positive reaction to CD68 and S-100 protein, whereas reaction to CD1a was negative. These findings allowed us to diagnose RDD.

During hospitalization, chest computed tomography (CT) was also performed, confirming the presence of right hilar adenopathy (18 × 14 × 20 mm), which we considered as another nodal localization of the disease. Abdominal CT scan was negative.

The patient was discharged after 14 days, with the prescription of oral prednisone at a standard dosage of 1.5 mg/kg/day. Therapy was gradually tapered and continued for overall 40 days. Clinical follow up showed an important reduction of both parotid swelling and cervical lymphadenopathy. The child was asymptomatic, with normal blood tests. In particular, CRP and ESR resulted negative after 20 days from the beginning of therapy. This was the first blood check performed after discharge, given that the child was asymptomatic and the clinical picture was improving.

Twenty days after suspension of oral steroid therapy, an MRI was repeated demonstrating a decrease in size of the right intraparotid mass (28 × 20 mm).

**Fig. 1:**
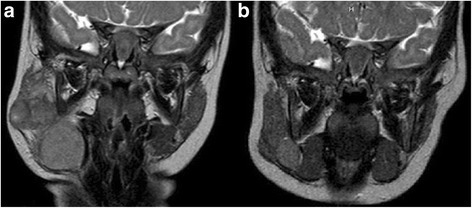
MRI of the face and neck findings. Evidence of a solid mass attributable to lymph nodes, entirely occupying right parotid cavity (**a**), with marked improvement on second control after prednisone course (**b**)

Left parotid lesions and all the other cervical lymphadenopathies previously reported were also reduced (Fig. 1).

**Fig. 2:**
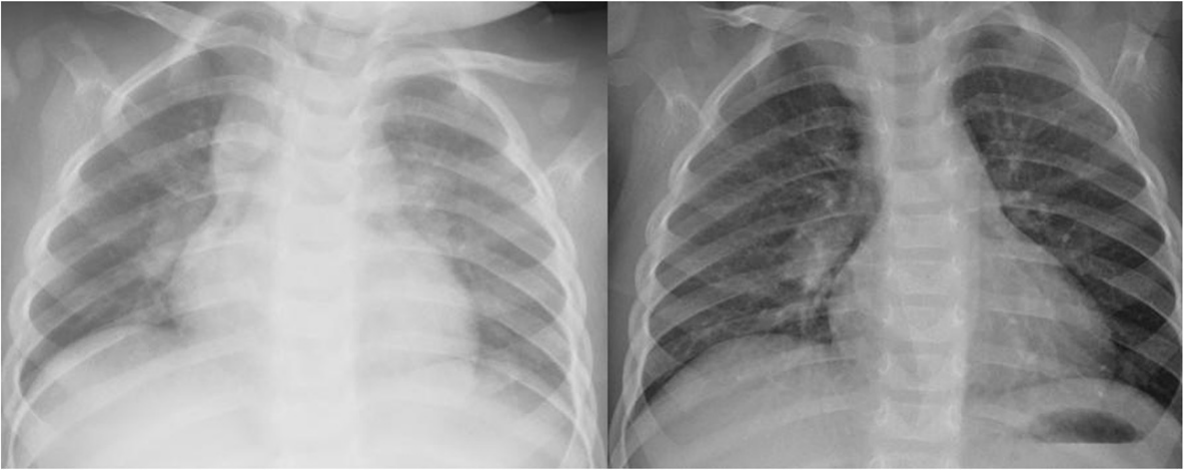
chest x-rays findings. Evidence of right mediastinal enlargement (*figure on the left*) on first control, with disappearance on second evaluation after prednisone course (*figure on the right*)

During follow-up, we witnessed a progressive dimensional decrease of the lesions. The right mediastinal enlargement shown on the first chest radiograph had already disappeared at first checkup visit (Fig. 2). Right parotid swelling disappeared, with US at 4 months after therapy interruption showing residual lymphadenopathies of 16 mm in the right parotid and 23 × 12 mm in the right part of the neck. We therefore decided to continue with a wait-and-see approach, with periodic clinical and US controls. Twelve months after the therapy discontinuation, the child was completely asymptomatic, with minimal residual lymph nodes within both right parotid and cervical area. The “CARE” checklist is available as Additional file 1. This child’s clinical course is summarized in the accompanying supplemental time-line file (Additional file 2).

## Discussion

Systemic form of RDD is associated with various diseases or factors involving the immune system, such as cases discovered at the time or after the diagnosis of various forms of leukemia or lymphoma, or even following bone marrow transplant for precursor-B acute lymphoblastic leukemia [6]. Lu et al. reported 4 cases in which RDD and malignant lymphoma were identified in the same lymph node biopsy specimen [7].

As in our case, the presence of EBV or HHV-6 in histological samples has also been reported [8]. Nevertheless, no strong association can be found between one of these factors and the occurrence of RDD.

However, it can be hypothesized that the disease may be triggered by a generic immunological stimulus, which could result in the accumulation and activation of histiocytes [9].

In our case, considering the presence of EBV in the specimen and the serological pattern indicating a recent infection (even though EBNA-IgG can be negative in a small percentage of cases), it is possible that the virus was the immunological trigger for the disease.

Cervical lymphadenopathy is present in over 90 % of patients affected by RDD. Typically, it is painless, bilateral and frequently massive, with single nodal elements measuring up to 6 centimeters [5]. However, practically any group of lymph nodes can be involved in RDD. Axillary and inguinal lymph nodes are enlarged in 38 and 44 % of cases, respectively. Mediastinal and hilar nodes are involved in approximately 30 % of patients. In a minority of cases, retroperitoneal lymph node localizations have also been described [5]. Except for cervical nodes, the dimension of adenopathy in the other sites is usually smaller. In our case, a non-massive cervical adenopathy was present, associated with enlargement of right hilar nodes and bilateral intraparotid nodes.

Extranodal involvement has been documented in 43 % of cases [10]. The most commonly affected sites are the upper respiratory tract (nasal cavities and paranasal sinuses), skin, eyes and retro-orbital tissue and bone tissues. Salivary glands and central nervous system are less frequently involved. Localizations of RDD in lungs, urogenital and gastrointestinal tract, breast, thyroid, and even heart have also been reported [9, 11–13].

The onset of the disease is generally subtle, with an average of 3–6 months between the beginning of signs and symptoms and the diagnosis. Non-specific systemic symptoms can be present, including fever, malaise, weight loss, and night sweats. They are more frequent in case of an important nodal involvement, but cases of patients with systemic symptoms in which RDD was purely extranodal have been reported [10]. In case of extranodal localizations, the clinical picture will depend on the affected organ or apparatus. In our case, the only systemic symptom was the low-grade fever, appearing approximately 40 days after the first observation of the parotid swelling.

Laboratory findings in RDD are non-specific. The most frequent features include leukocytosis, elevated ESR and CRP levels, and polyclonal gammopathy. Normochromic normocytic anemia and elevated serum ferritin levels have also been described. Less common laboratory abnormalities include positivity for rheumatoid factor and antinuclear antibodies, and a reversal of the CD4/CD8 ratio in peripheral lymphocytes [13]. There are also reports on RDD complicated by the development of autoimmune hemolytic anemia, especially in children, for which the pathogenetic mechanism is not known [14].

Imaging exams may be helpful in differential diagnosis, but they are not pathognomonic. In MRI studies of patients affected by RDD the areas involved were generally T1 isointense, T2 isointense, diffusion isointense to gray matter, and intensely enhancing with gadolinium, whereas in CT images the lesions were hyperdense to gray matter and intensely enhancing [15]. Ultrasound can also be helpful, especially in neck involvement, to see if lymphadenopathy is either focal or diffuse and in follow up, being a noninvasive and relatively rapid exam.

Definitive diagnosis can only be made by histological analysis of affected lymph nodes or tissues. The association between emperipolesis, defined as the presence of phagocytized cells (mainly lymphocytes, but also plasma cells, neutrophils or erythrocytes) in a histiocyte, and a typical immunohistochemical pattern characterized by positivity for S-100 protein and CD68 antigen and negativity for CD1a antigen, is diagnostic for RDD. Emperipolesis alone is highly suggestive of the disease, but it can also be found in Langerhans cell histiocytosis (LCH), autoimmune hepatitis, lymphoma and rhinoscleroma, a chronic granulomatous bacterial disease affecting the nose and rarely the upper respiratory tract, caused by Klebsiella rhinoscleromatis, a subspecies of Klebsiella pneumoniae [14, 16]. Another characteristic is the absence of Birbeck granules, which are instead typical of LCH. LCH and RDD can be also distinguished by CD1a pattern, being nearly always expressed in LCH. The clinical course of RDD is generally benign, with spontaneous complete resolution in most cases, especially if the disease affects mainly lymph nodes. However, locoregional recurrence of RDD and even dissemination can be possible, particularly in forms with extranodal involvement. There have also been reports of deaths directly caused by RDD, even in children, especially in severe extranodal forms, with involvement of CNS, kidneys or respiratory tract [17].

There is no consensus for the best therapeutic approach. An initial wait-and-see approach may be the best option, given the fact that RDD is often self-limiting.

However, in case of significant extranodal disease and/or compression of vital organs by massive lymphadenopathy, prompt therapy may be indicated.

When possible, surgery probably represents the best solution, being curative if resection is complete. Recurrence after surgical therapy is very rare and limited to incomplete debulking and multiorgan involvement [4].

Medical therapeutic options include corticosteroids, antibiotics, antiviral agents, chemotherapy, and radiotherapy. However, no universally accepted treatment guidelines exist. Several Authors reported successful treatments using radiotherapy and/or corticosteroids in cases of recurrences or incomplete resection, but this strategy was not always effective. In our case, given the young age of the patient and the apparently rapid progression of the disease, we decided to treat the child using corticosteroids, obtaining an important dimensional reduction of hilar, cervical and parotid glands adenopathy, the resolution of systemic symptoms, and normalization of blood tests. Moreover, we chose to treat the patient with a relatively short course of prednisone therapy at a standard dosage of 1.5 mg/kg/day, obtaining a good clinical response and no adverse effects.

In order to establish whether there could be a more effective medical approach in the management of RDD, in particular in children, we reviewed the literature through both PubMed and Embase network using *Rosai Dorfman disease*, *sinus histiocytosis with massive lymphadenopathy* and *child* as keywords and selecting all the available pediatric manuscripts published between 2004 and 2014. In addition, we reviewed references cited in all selected manuscripts to identify additional reports of pediatric RDD. We did not include cases in which the surgery was used as the only therapeutic approach (mainly isolated intracranial forms of RDD). We selected also all cases in which there was an apparent involvement of the salivary glands at presentation, as in our patient, including 2 reports in which there was no information on clinical outcome (Table 1) [18, 19]. In one of these two cases, only surgery was performed, but we decided to include it in the Review, given the involvement of the salivary glands as in our case. In Table 2 we report a summary of all other clinical reports found. Correlation between the clinical outcome and the most effective therapy used in single cases was obtained with the chi-square test. *P* values of less than 0.05 were considered statistically significant. Complete regression of RDD, improvement and clinical stability of the disease were considered as clinical outcomes in our analysis. The only case in which the disease led to death was not included in the statistical analysis regarding the connection between outcome and therapy, as well as 3 cases in which outcome was not precise [17–20].

**Table 1:** Pediatric cases of RDD with involvement of salivary glands

Age/sex	Clinical picture at presentation	Main lesion location	Systemic symptoms and/or abnormal blood tests at presentation	Nodal and extranodal involvement	Therapy and clinical evolution	Outcome	Ref.
10/M	Painless masses around parotid and submandibular glands.	Parotid and submandibular glands bilaterally.	None	Apparently both nodal and extranodal	None	Symptom-free	[21]
9/M	Masses around submandibular glands	Submandibular glands bilaterally.	None	Apparently both nodal and extranodal	None	Symptom-free	[21]
11/M	1 year history of painless bilateral neck swelling.	Submandibular and parotid glands bilaterally.	None	Both nodal and extranodal	Surgery	Not reported	[18]
17/F	Bilateral parotid enlargement and cervical lumps localized in the submandibular region.	Mass at left common carotid artery, descending aorta down to the renal artery; MRI finding of bilateral lesions in knee and ankle.	High CRP and ESR, hypergammaglobulinemia.	Both nodal and extranodal	None	Not reported	[19]
12/F	1-month history of enlarging and painless submandibular lymphadenopathy.	Parotid and submandibular glands.	Recurrent fever 2 months before presentation; high ESR.	Nodal	None	No recurrence after 28 month of follow-up.	[28]

**Table 2:** Summary of all other pediatric cases of RDD described between 2004 and 2014 (our case and cases in which only surgery was used were not included; i.e. 33 cases)

Systemic symptoms	Fever	Anemia	Fatigue	None	Not mentioned
# of cases	3	5	1	10	9
Ref.	[25, 29, 30]	[3, 24, 27, 29, 31]	[32]	[17, 20, 24, 28, 30, 32–35]	[6, 17, 23, 24, 26, 36–38]
Lesion location	Lymph nodes	Bone	Brain	Other	
# of cases	18	8	5	6	
Ref.	[17, 22, 25, 27–36, 39, 40]	[6, 22, 24, 34, 37, 40]	[20, 24, 26, 38, 39]	[3, 17, 23, 30, 32, 41]	
Successful of main treatment	Corticosteroids	Chemotherapy	Corticosteroids + chemotherapy	Others	None
# of cases	6	7	5	5	10
Ref.	[3, 23, 30, 33, 36]	[6, 22–24, 32, 37, 40]	[17, 24, 34, 39]	[20, 25–27, 38]	[17, 24, 29, 31, 28, 35, 41]
Outcome	Complete regression	Partial regression	Clinical stability	Symptoms free (no precise information on disease outcome)	Death
# of cases	11	12	6	3	1
Ref.	[6, 22, 25–27, 28, 33, 35, 36, 38, 39]	[3, 23, 24, 29–32, 34, 39, 40]	[17, 24, 37]	[17, 29, 41]	[20]

Overall, we found 35 pediatric cases of RDD in literature (36 including our case, which was considered in the analysis). Mean age of the children described in the manuscripts was 8.79 years [standard deviation (SD) 4.26, minimum 14 months, maximum 17 years], with a clear prevalence in males (68.6 % vs 31.4 %), as confirmed by other studies. During the period we considered, our case was the youngest reported. Almost half of the patients had both nodal and extranodal involvement (17). Eleven children had purely nodal RDD, whereas the remaining 8 patients had only extranodal localizations. There was no correlation between the type of RDD and clinical outcome. However, we found a significant difference between the mean age of children and the type of RDD (Table 3), with younger children being those most frequently affected by purely nodal RDD. To our knowledge, this epidemiological finding was not previously reported. In the analysis, we considered the age at the time of first diagnosis, excluding 2 cases in which this element was not reported [21].

**Table 3:** Chronological age at diagnosis of different forms of RDD (nodal, extranodal, both nodal and extranodal). Data are reported ad mean ± SD; Kruskal-Wallis test

	Nodal RDD	Extranodal RDD	Nodal and extranodal RDD	*p*
Age (yrs)	5.83 ± 4.89	10.0 ± 2.68	9.43 ± 3.54	0.018

Considering the medical therapeutic approaches, there was a statistically significant difference correlating the most effective strategy used in single cases and the clinical outcome (p 0.033). Prednisone and prednisolone were the most used single drugs. In 6 cases they were the only treatment used, with complete regression in 2 children and clinical improvement in the other 4 cases. Chemotherapy was shown to be an effective strategy, with complete regression in 4 patients, clinical improvement in 5 and clinical stability in 1 subject. However, it has to be said that the chemotherapic agents reported were various, giving different results. For example, 2-chlorodeoxyadenosine (2-CDA) was found to be very effective in a case in which prednisone, 6-mercaptopurine and vinblastine were used before, without clinical response [22]. Nevertheless, this drug was ineffective in other reports [23, 24]. In 3 children, the successful treatment consisted of another medical approach (radiotherapy, rituximab in association with prednisone, α-interferon) [25–27]. However, in our opinion these strategies cannot be recommended as first-line treatment, as they have been cited only in single case reports. Finally, no treatment was used in 9 cases, with complete regression in 2 children, clinical improvement in 3 and clinical stability in 4 subjects. This means that, even if RDD was reported to have a benign course in most of the cases, medical therapy may hasten remission, given the fact that treated children had a significant higher percentage of improvement (Table 4). Moreover, complications of the disease are not so infrequent. That is why, in our opinion, it would be safer, if complete surgical debulking is not possible, to proceed with a therapeutic medical approach, with the goal of hastening regression of the disease and avoiding complications. In light of the data presented, with all the limits related to a review of case reports, in our opinion it would be safe and worthwhile to start with a course of oral steroid therapy as a first-line approach. In case of no clinical response, there is still no agreement on what might be the best treatment. Chemotherapy was shown to be effective; however, clinical response to the same protocols did not appear to be constant. Alternative therapies were effective in some cases, but they should be tested in more subjects.

**Table 4:** Type of approach and clinical outcome (*p* = 0.033, chi-squared test). Cases in which only surgery was used and/or outcome was death or not precise were not included; i.e. 32 cases

Outcome/Therapy	None	Steroid	Chemo	Others
Complete regression	2	3	4	3
Clinical improvement	3	4	5	1
Clinical stability	5	0	2	0

## Conclusions

To our best knowledge, this is the first RDD case that involves both parotid and hilar lymph nodes in a toddler, apparently without extranodal localization.

Hilar adenopathy biopsy was not performed, but the unilateral pattern, the absence of any other clinical reason that could explain the mediastinal enlargement and the prompt resolution after oral steroid therapy are in our opinion 3 strong factors that confirm the adenopathy as another nodal localization of RDD.

Another interesting factor is that progression of the disease was quite rapid, differently from other case reports in which the development of parotid swelling was more gradual.

A short course of prednisone was shown to be an effective treatment in our case.

RDD should be included in differential diagnosis of parotid swelling even in children.

A medical approach should be considered in all pediatric cases of RDD.

## Consent

Written informed consent was obtained from the patient’s parents for publication of this Case report and any accompanying images. A copy of the written consent is available for review by the Editor of this journal.

## Additional files

Additional file 1: CARE Checklist of information to include when writing a case report. (DOC 1546 kb) Additional file 2: Timeline. (PPT 138 kb)

## Abbreviations

2-CDA: 2-Chlorodeoxyadenosine
CMV: cytomegalovirus
CNS: central nervous system
CRP: C-reactive protein
CT: computed tomography
EBV: Epstein - Barr virus
ESR: elevated erythrocyte sedimentation rate
HHV-6: herpesvirus
LCH: Langerhans cell histiocytosis
MRI: magnetic resonance imaging
MTX: methotrexate
RDD: Rosai-Dorfman disease
SD: standard deviation
US: ultrasonography
