# Supplementary material for: Unusual presentation of Rosai-Dorfman disease in a 14-month-old Italian child: a case report and review of the literature
Source: BMC Pediatr. 2016 May 3;16:62. doi: 10.1186/s12887-016-0595-9 (PMC4855344; doi:10.1186/s12887-016-0595-9)
Supplement: Additional file 2: — Timeline. (PPT 138 kb) [file 12887_2016_595_MOESM2_ESM.ppt]

## Slide 1
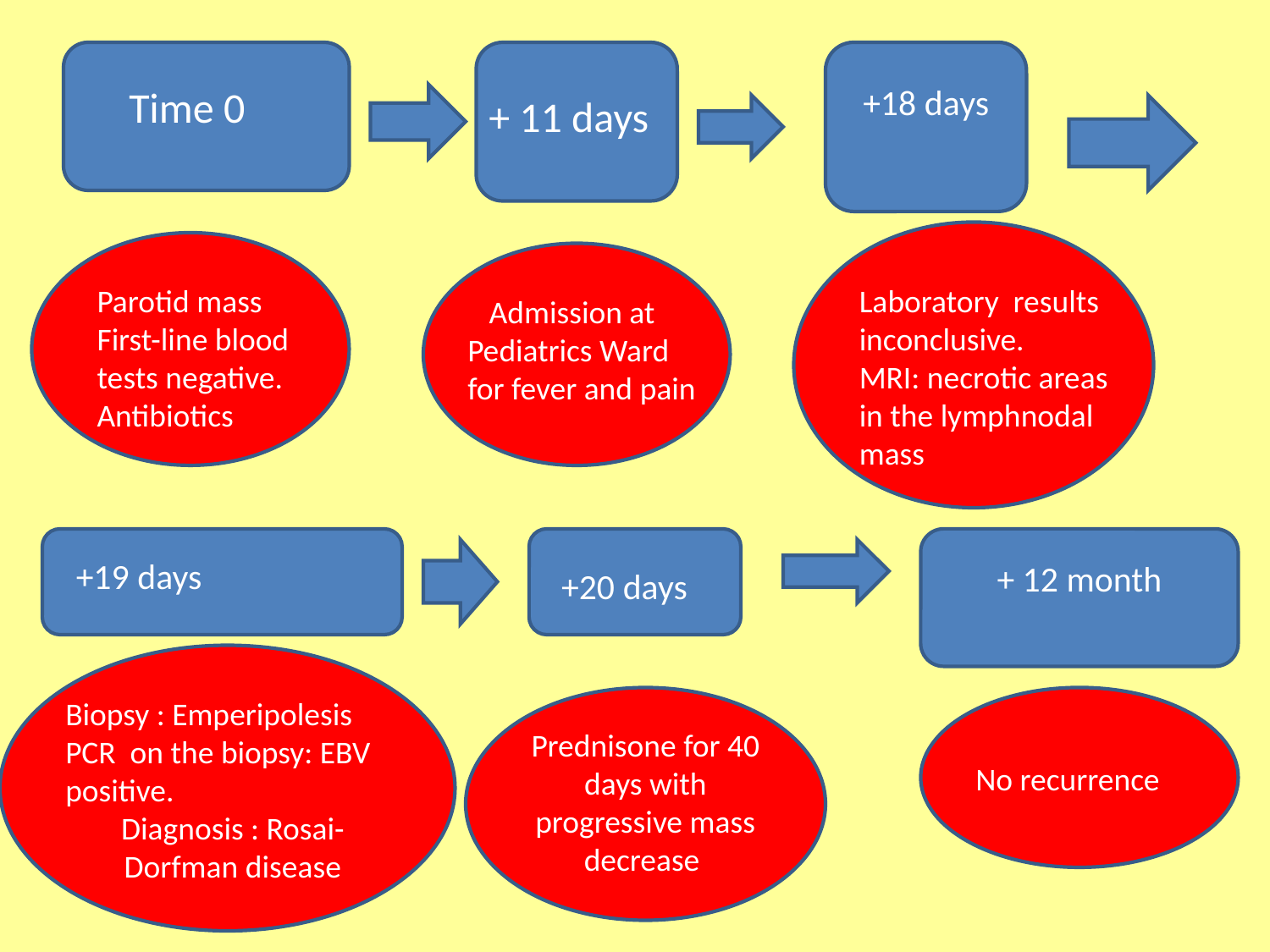

Time 0
+18 days
+ 11 days
Parotid mass
First-line blood tests negative.
Antibiotics
Laboratory results inconclusive.
MRI: necrotic areas in the lymphnodal mass
 Admission at Pediatrics Ward for fever and pain
+19 days
+ 12 month
+20 days
Biopsy : Emperipolesis
PCR on the biopsy: EBV positive.
Diagnosis : Rosai-Dorfman disease
Prednisone for 40 days with progressive mass decrease
No recurrence
